# The role of food security in increasing adolescent girls’ agency towards sexual risk taking*:* qualitative findings from an income generating agricultural intervention in southwestern Kenya

**DOI:** 10.1186/s12889-021-12051-6

**Published:** 2021-11-06

**Authors:** Maricianah A. Onono, Gladys Odhiambo, Lila Sheira, Amy Conroy, Torsten B. Neilands, Elizabeth A. Bukusi, Sheri D. Weiser

**Affiliations:** 1 Kenya Medical Research Institute, Center for Microbiology Research, P.O. Box 19669-40123, Kisumu, Kenya; 2 Department of Medicine, Division of HIV, Infectious Diseases, and Global Medicine, University of California San Francisco, San Francisco, USA; 3 Division of Prevention Science, Center for AIDS Prevention Studies, University of California San Francisco, San Francisco, USA

**Keywords:** Food insecurity, Sexual risk taking, Adolescents, Sexual agency, Structural interventions, Africa

## Abstract

**Background:**

Food insecurity is an important underlying driver of HIV risk and vulnerability among adolescents in sub-Saharan Africa. In this region, adolescents account for 80% of all new HIV infections. The primary purpose of this analysis is to understand perceived mechanisms for how a multisectoral agricultural intervention influenced sexual risk taking among HIV-affected adolescents in southwestern Kenya.

**Methods:**

We conducted semi-structured, individual interviews with 34 adolescent-caregiver dyads who were participants in *Adolescent Shamb*a *Maisha* (NCT03741634), a sub-study of adolescent girls and caregivers with a household member participating in the *Shamba Maisha* trial (NCT01548599), a multi-sectoral agricultural and microfinance intervention. Interviews were audiotaped, transcribed, translated, and analyzed using framework and interpretive description analysis methods.

**Results:**

Adolescents receiving the *Shamba Maisha* intervention described no longer needing to engage in transactional sex or have multiple concurrent sexual partners as a way to meet their basic needs, including food. Key mechanisms for these effects include greater sexual agency among adolescent girls, and increased confidence and self-efficacy in overcoming existing reciprocity norms and sexual relationship power inequity; as well as staying in school. The intervention also increased caregiver confidence in talking about adolescent sexual reproductive health issues. In contrast, driven primarily by the need for food and basic needs, girls in the control arms described engaging in transactional sex, having multiple sexual partners, being unable to focus in school, getting pregnant or becoming HIV infected.

**Conclusion:**

These findings emphasize the need to address food insecurity as a part of structural interventions targeting adolescent HIV risk in low-resource countries. We recommend that future interventions build upon the *Shamba Maisha* model by combining sustainable agricultural production, with household level interventions that deliberately target gender norms that contribute to unequal power dynamics.

**Supplementary Information:**

The online version contains supplementary material available at 10.1186/s12889-021-12051-6.

## Background

Adolescent girls and young women (AGYW) in sub-Saharan Africa (SSA) are at a high risk of HIV acquisition. In east and southern Africa, AGYW account for 80% of all new HIV infections [1, 2]. Food insecurity, defined as “the limited or uncertain availability of nutritionally adequate, safe foods, or the inability to acquire personally acceptable foods in socially acceptable ways” [3], is an important underlying driver of HIV risk and vulnerability among AGYW [4–6]. Food insecurity is associated with higher sexual risk taking behaviour, sexually transmitted infections (STIs), and even prevalent HIV [7–9]. Food insecurity also impedes appropriate healthcare-seeking behaviour for reproductive health. Adolescents who are affected by HIV (defined as either having lost one or both parents to HIV/AIDS or residing with an adult caregiver chronically ill due to HIV/AIDS) are at particularly high risk of living in food insecure and impoverished households and exhibiting high sexual risk-taking behaviour.

There is increasing international recognition that improving food security and reducing poverty are essential for a successful global response to the HIV epidemic [10, 11]. Structural interventions that consider the biomedical, behavioural and socio-economic risk factors of HIV transmission potentially have an important role in confronting the complex risk environment underlying high rates of HIV infection in AGYW in SSA. Extant literature has demonstrated that HIV-affected adolescents who have access to a comprehensive economic strengthening intervention comprising matched savings accounts, financial management workshops, and mentorship (intervention arm) had better mental health functioning [12], self esteem and physical health, [13] educational outcomes, [14, 15] HIV prevention attitudes [15] and reduced sexual risk taking intentions [16]. Yet, these existing structural interventions have not demonstrated the anticipated benefits of reducing the rate of safer sex and have at times increased AGYW’s exposure to physical harm, sexual abuse, and coercion. One potential explanation for this could be that these interventions employ an individual-centred approach that focuses on the AGYW alone without supporting their households [17–26]. Based on the unintended consequences of economic strengthening interventions for young women, it is clear that there may be hazards of promoting individual-centered approaches with girls and young women in precarious economic environments without also ensuring adequate family and/or social support [20, 27–29].

Household-level interventions that target both poverty and food insecurity may offer many advantages over individual-centred approaches in reducing AGYW sexual risk taking. This is because household-level interventions address the context for the risk behaviour [30]. Studies show that dyad, family and community domains explain more variance in risk outcomes than traditional individual level factors alone [31]. In fact, regarding health, STIs and pregnancy risk, there have been calls for research that go beyond the individual and address factors located within a person’s social environment (e.g., family) [30, 31]. Agricultural interventions, such as the introduction of home or community gardens or the provision of agricultural training – have the potential to address the two core issues of food security and poverty and improve health outcomes for the family as a whole. Moreover, by empowering parents/guardians with agricultural and financial skills, such interventions address the issue of sustainability beyond the project period, since households are able to continue farming long after the project has ended.

There are limited qualitative data exploring potential mechanisms linking interventions at household level food security and poverty alleviation with sexual risk taking among AGYW [32]. It is important to understand the individual and combined lived experience of AGYW and their guardians in households receiving such interventions so as to complement the quantitative evidence and help contextualize intervention findings. The main objective of this study was to investigate the perceived effects and underlying pathways of a multisectoral income generating agricultural intervention on sexual risk taking among HIV-affected adolescents and their caregivers in southwestern Kenya. In this paper we demonstrate that *Shamba Maisha* intervention had an impact on four key areas related to adolescent sexual and reproductive health: 1) decreased transactional sex, 2) decreased multiple concurrent sexual partners 3) increased engagement in school and subsequent commitment to delay engagement in sex, and 4) increased caregiver confidence discussing SRH with adolescents. Providing both guardian and adolescent perspectives allowed us to highlight relational, individual, and structural factors that influenced the enactment of safer sex among adolescent girls.

## Methods

### Research setting and collaboration

Homabay, Kisumu, and Migori Counties in southwestern Kenya have high rates of both HIV and food insecurity [33, 34]. At the end of 2018, prevalence rates of HIV were 19.6%, 17.5% and 13% in Homabay, Kisumu and Migori respectively, making up nearly half of HIV cases nationwide. Furthermore, in this region AGYW are nearly four times as likely as their male counterparts to be infected (11% vs. 3%) [33]. The region also has the highest teenage pregnancy rate of 27% [35]. This southwestern region is one of Kenya’s most vulnerable regions to food insecurity with approximately 40% of households report lacking food or money to purchase food [34]. The proposed study was conducted in parallel with and linked to the parent study, a matched-pair cluster randomized controlled trial (RCT) of a multisectoral agricultural intervention among HIV-infected farmers on ART, designed to determine the impact of the intervention on adults’ nutritional and HIV clinical outcomes known as *Shamba Maisha*. *Shamba Maisha,* as has been previously described in several publications, was designed to improve household food security and household wealth [36, 37]. It had three components: a) KickStart irrigation pump; b) agricultural training on sustainable farming practices and c) a loan (~$175) from a well-established Kenyan finance organization for purchasing agricultural implements and commodities and training in financial management.

### Study population

The study population was adolescent girls aged 13–19 and their primary guardians enrolled in the Adolescent *Shamba Maisha *study. The study enrolled 241 dyads from October 2018–December 2019. The adolescent girl had to live in the same household as a *Shamba Maisha* participant in either the intervention or control group and the caregiver was not necessarily the index *Shamba Maisha* participant. The primary guardian is defined as the parent/guardian who is primarily responsible for preparing food for the adolescent and seeking healthcare (i.e., for illness) for the adolescent when needed.

### Recruitment strategy

At both intervention (*N* = 8 sites) and control sites (N = 8 sites), we purposively recruited and enrolled 34 dyads comprising adolescents 13–19 years old who were enrolled in the *Adolescent Shamba Maisha* sub-study with their primary caregiver. Households within the compounds were eligible if they had (1) an adult who was a participant in the parent study, (2) > 1 adolescent aged 13–19 years old who was not born with HIV, and (3) the adolescent had a parent/primary guardian age > 18 years old who resided in the compound. The parent or guardian of an eligible adolescent was eligible if they were the primary caregiver for the adolescent, and were either a participant in the parent study *or* resident in compound of participant in the parent study. We excluded married adolescents, those born with HIV or known sero-converters prior to the start of *Shamba Maisha*, and those who were themselves heads of households, including those ages 18 and 19 who were enrolled in the parent study. Adolescents who had inadequate cognitive and/or hearing capacity to complete planned study procedures were also excluded.

### Data collection

Semi-structured in-depth interviews were conducted with caregiver-adolescent dyads between April and December 2019 (interview guides used in the study are available as additional files 1, 2, & 3). After collecting demographic information on gender, sex, education, parity and marital status, trained trilingual field workers conducted interviews in English, Dholuo or Kiswahili. The interviews followed a semi-structured in-depth interview guide with open-ended questions and probes structured to elicit reflections on intervention impact, finances, life situation, food security, and sexual health. Questions related to sexual behaviour explored sexual relationships, sexual practices including use of condoms, experiences of transactional sex and concurrent sexual partners and factors that impacted decision making around partner selection. Questions were similar between adolescents and caregivers, lasted approximately 90 min and were digitally recorded and transcribed. Adolescents and caregivers were interviewed separately and asynchronously in a private location, in the health facility or home, at the discretion of the participant. The initial plan was to interview at least 40 dyads; however, saturation was reached at the 34th dyad. Two dyads (one intervention and one control) were approached but they were not willing to participate. The control dyad did not want to participate because they felt they had not benefited from the intervention and so contributing to the study was a waste of their time, while the intervention dyad, the adolescent had travelled out of the study area and were not willing to come back for data collection.

### Data analysis

Interview transcripts were transcribed and translated into English for analysis. The data was analysed using ‘Framework’ [38] and ‘Interpretive description’, [39] both widely used analytical tools that complement each other. Framework analysis involves indexing all verbatim text, charting information from each transcript on to a series of thematic matrices. Choice of thematic headings were guided by both the core concepts emerging out of the data using an open coding approach [40] and by theoretical concepts from the design process [39]. In the tradition of interpretive description, a rigorous process, alternating between interview transcripts, codes, discussion notes, field notes and relevant empirical and theoretical literature on the topic leading to themes was employed. The primary interviewer coded all transcripts using the qualitative text management software Dedoose. Double coding by the lead Kenyan investigator took place at predetermined intervals (every fourth transcript; *n* = 17), with discrepancies discussed and resolved by consensus to validate the codebook and maximize coding reliability. Throughout data collection and analysis, we practiced reflexivity by continually examining our own biases, preferences, and theoretical perspectives and how those factors played a role in our understanding and interpretation of the processes and data we were analysing [41]. Selected quotations were chosen to illustrate key themes and sub-themes. All names accompanying quotations are pseudonyms.

## Results

### Participants characteristics

We conducted a total of 68 in-depth interviews among 34 adolescent-caregiver dyads (46 intervention and 22 control interviews). Adolescent age ranged from 13 to 19 years. A majority of caregivers (52%) had at least five children (3–11 children). Most caregivers were female (29 female and 5 male). Table 1 provides additional sociodemographic data for the adolescents’ caregivers. All adolescents were single, 11 were in primary school, 22 in secondary and one in tertiary level. At the time of the interview, six adolescents self-reported being pregnant or having ever been pregnant (five in the control arm) and two adolescents from the control arm self-reported as being HIV positive. Four key themes emerged linking food insecurity with risky sex, which were mitigated by the *Shamba Maisha* intervention. These include 1) transactional sex, 2) multiple concurrent sexual partners 3) partner selection 4) decision to delay engagement in sex.

A. Linkages between food insecurity and sexual risk taking among adolescent girls

**Table 1:** Adolescent girls’ caregiver characteristics stratified by intervention group

	Control	Intervention
n	11	23
**Age** (median [Q25, Q75])	40 (38, 50)	43 (37, 49)
**Sex** (%)		
Male (1)	1 (9.1)	3 (13.0)
Female (2)	9 (81.8)	20 (87.0)
NA	1 (9.1)	0 (0.0)
**Any education** (%)		
Yes, but not currently	10 (90.9)	22 (95.7)
No	0 (0.0)	1 (4.3)
NA	1 (9.1)	0 (0.0)
**Years of education** (median [Q25, Q75])	8 (8, 11)	7 (7,9)
**Highest level of education achieved (%)**		
Some primary (2)	4 (36.4)	10 (43.5)
Primary (3)	3 (27.3)	3 (13.0)
Some secondary (4)	3 (27.3)	4 (17.4)
Secondary (5)	0 (0.0)	3 (13.0)
Certificate (7)	0 (0.0)	1 (4.3)
Diploma (8)	0 (0.0)	1 (4.3)
NA	1 (9.1)	1 (4.3)
**Relationship status (%)**		
Married (1)	6 (54.5)	15 (65.2)
Widowed (4)	3 (27.3)	7 (30.4)
Divorced (5)	1 (9.1)	1 (4.3)
NA	1 (9.1)	0 (0.0)
**HIV Status** (%)		
HIV -	0 (0.0)	2 (8.7)
HIV +	10 (90.9)	21 (91.3)
NA	1 (9.1)	0 (0.0)

During the interviews, intervention participants reminisced over previous struggles in obtaining food and further highlighted how the *Shamba Maisha* intervention had enabled them to obtain food in more acceptable manners and reduced their engagement in sexual risk taking. On the other hand, most control participants described experiencing financial hardship and food insecurity. Prior to the intervention and in the control arm, girls engaged in high-risk sexual behaviour such as transactional sex, multiple partnerships, and condomless sex. This high-risk sexual behaviour among the adolescent girls took place in different types of sexual relationships. The girls had sex in exchange of money, clothes, food and basic necessities like sanitary towels, soap and body lotions. For some girls, these gifts were the primary reason to be in the relationship, while for others, the gifts were an additional incentive to maintain relationships and engage in sex, often unprotected. At times, it was not so much the lack of food that prompted the sex, but desire for more dietary diversity in form of snacks. Below a participant from the control cohort highlights how she got variety of food from her partner.

*At times he can give me 200 shillings after having sex … My boyfriend has also been buying me chips, soda and cakes* – Apondi, Adolescent, Control, 17 years

Some also described the phenomenon of “Jaboya”, where girls felt forced to exchange sex in order to get fish for themselves and their family. This put them at risk for sexually transmitted infections and gender-based violence. For example, a caregiver in the intervention arm describes how this played out for one of her neighbors.

*This I say based on a story I happen to have witnessed. There is a 12-year-old girl who left home going for sport activities, later when her mother was looking for her, she stayed hidden in the house of a boy. When found the boy who hid her claimed that he has been giving the girl fish, which her family has been feasting on. From this experience, you have to ask questions whenever your child brings you something like food often; don’t just eat without knowing where your child got money to buy the food*. -Nyagot, Female Caregiver, Intervention.

B. **Perceived impact of** ***Shamba Maisha***

In general, participants perceived that the *Shamba Maisha* intervention had an impact on four key areas related to adolescent sexual and reproductive health: 1) decreased transactional sex, 2) decreased multiple concurrent sexual partners 3) increased engagement in school and subsequent commitment to delay engagement in sex, and 4) increased caregiver confidence discussing SRH with adolescents.

1. **Decreased need for transactional sex as a food procurement strategy**

Participants described that the *Shamba Maisha* intervention reduced the need for transactional sex through addressing reciprocity norms, partner selection as well as sexual relationship power equity. This was due to the fact that the intervention empowered caregivers to provide for the needs of adolescent girls and this made them less vulnerable to transactional sex.

#### Reciprocity norms and partner selection

Adolescent girls described that having food and financial security from participation in *Shamba Maisha* minimized their need to engage in reciprocity; i.e., the idea that if you receive something from someone you should give something in exchange. Repeatedly in the interviews, adolescent respondents stated that having no food at home made them give in to partners’ sexual demands as a way to pay back favours that they had received, which included being given food.

*The main reason as to why I was with him is the baby [younger sibling] who needed food then and I also needed food (before Shamba Maisha). There were times the baby would cry because of hunger before our mother came back from her hustle. I would then call my boyfriend and tell him that we do not have food and he would send me money. If he sends Kshs. 1000, I would go to the shop, buy flour and other food stuffs for the family to eat during lunch time-* Awiti, Adolescent, Intervention, 19 years

Adolescents from the intervention group felt that their improved family’s economic status gave them the agency to eschew transactional relationships, for example, one girl, whose father’s monthly salary had been greatly boosted by regular income from the intervention, proudly stated:

*When your parents provide for your needs then you will not have the urge to have sex for economic gains. You know girls need sanitary towels and if my father does not provide them for me then I have to think of ways of getting them. The next option is selling my body so that you can get sanitary towels. I am not at risk of doing such things because my father provides for all my needs. Therefore, I do not have any pressure to have sex. -* Lily, Adolescent, Intervention, 17 years

On the contrary, girls in the control arm deliberately selected partners based on their ability to provide and fill the gap they had considering what their parents were able to provide for them. Similar to the adolescent girl above, for example, one participant in the control arm described how her siblings’ needs were always met before hers. In the interview she talks of being sidelined “(my dad) *always sidelined me in paying school fees yet he was paying for the other children. I would be sent away from school yet my siblings always stayed at school since their fees were paid for*.” Unfortunately, for this young girl, she self reported contracting HIV and was on medication at the time of the interview. She explained her reason for choosing a sexual partner to be based on the fact that he was providing for her needs, further strengthening the fact that lack of households’ essentials including food is one of the reasons for adolescents’ sexual risk taking.

*I: Why did you choose to have him as your boyfriend?*

*R: He was very close to me and whenever I lacked anything, he would provide for me.*

*I: What are some of the things he has provided for you?*

*R: He gives me things like body lotion, clothes and there is a time he also gave me some 100 shilling when I had been sent away for from school, the money was needed at school and I told him about it then he gave me the money. -* Achieng, Adolescent, Control, 15 years

Other adolescents explained that lack of food incentivized transactional sex among adolescents.

*Well, since they don’t have food in the house, they will feel the need to go and look for money by all means and maybe a boyfriend might present himself in the form of help and that’s how it begins, before you know it you are already having sex with him. The girl has to pay him in the form of sex. The girl has to have sex with him in return.* -Akoth, Adolescent, Intervention, 17 years

*... if you have the basic needs then you will be able to decline any money or food offers from a man and so you stay safe from sexual advances … I wouldn’t have a sexual partner because all the things he can provide for me such as food and money would be available at home-* Apondi, Adolescent, Control, 17 years

#### Gender equity: sexual relationship power equity

The participants reported that *Shamba Maisha* gave them a sense of empowerment and sexual relationship power equity within their relationships. Often dependence on male partners for food and other resources disempowers women by making it difficult to exercise control in sexual decision-making. Participants described a positive shift in sexual relationship power dynamics. In the past, they felt passive and often deferred to their partners’ desires, whereas now they felt empowered to be more assertive. This contributed to improvement in relationship dynamics, and adolescent girls also felt more empowered in their sexual relationships. For example, one adolescent girl described how the primary reason she had maintained her partners was to get food for her and for the family. This was not someone she wanted to be with, but he was able to provide for her younger brother and for herself. However, since her family joined the *Shamba Maisha* intervention, she no longer needed to do so because they now had access to food and resources after the *Shamba Maisha* intervention.

*I now have sex when I am ready to because I am confident. Moreover, I can face my man and tell him that I want sex. Before, I could not even talk …* Awiti, Adolescent, Intervention, 19 years

2. **Decreased need for multiple concurrent sexual partners**

Another key improvement described by adolescent girls is that after participating in *Shamba Maisha*, they felt a reduced need to have multiple sexual partners. Girls receiving the intervention were able to take control over their own sexuality and resist the need to have multiple partners. The girl below who started receiving a monthly stipend from her father after he enrolled in the intervention states that she has no temptation at all to have multiple boyfriends. She proudly explained:

*Because I will get food at home so I don’t have that urge to have several boyfriends so that if I go to one, he can give me some money to go buy food to feed my family or myself because I’m hungry, no … that has never happened! -* Akinyi, Adolescent, Intervention, 14 years.

In contrast, adolescents in the control arm expressed the need for multiple partners to meet a diverse set of needs. According to them, having many sexual partners meant having more economic gain as well as accessing a diverse diet. In the conversations below, the young girl had multiple “functional” sexual partners.

*I have two boyfriends … having two boyfriends mean more profit and so that’s why I decided to have two boyfriends... One can give me money then another one comes and gives me chips …* - Apondi, Adolescent, Control, 17 years.

Her mother also confirmed her behaviour of having many sexual partners.

*She is very promiscuous. Recently she spent two nights … She has sexual affairs with men* – Nyagem, Female Caregiver, Control

However, the girl who was visibly pregnant at the time of the interview points out that if she had enough food and basic needs, this probably would not have been the case.

*… If you have the basic needs then you will be able to decline any money or food offers from a man and so you stay safe from sexual advances … I wouldn’t have sexual partners if all the things they can provide for me such as food and money would be available at home* - Apondi, Adolescent, Control, 17 years

At the time of the interview, this girl was also recently diagnosed as being HIV-positive.

3. **Delay in sexual debut by keeping girls in school**

According to participants, *Shamba Maisha* contributed to a delay in sexual debut due to both improvements in food and financial insecurity. Food secure households used the surplus to pay for fees and keep girls in school. Being in school was highlighted as one the main reasons adolescents were able to delay having sex which then lowered their risk of getting HIV, STI and pregnancies.

*I want to complete my high school and move to campus so that I can be somebody in the future...When you have sex; you might contract diseases such as STDs, HIV and pregnancy. This can make you to drop out of school* – Rita, Adolescent, Intervention, 15 years

In addition, girls receiving the intervention perceived that education would bring financial autonomy through getting jobs, which in turn, would make them self-reliant and decrease their dependence on sex as a resource to support their families

*I am not ready (for sex) because I am still going to school, I haven’t got myself a job that I can help me care for my household.* – Awuor, Adolescent, Intervention, 17 years

This parent, talks about her daughter’s dreams while contrasting with those of her peers who are not in the intervention

*In today’s world, most girls become sexually active at very tender ages; in our area, some get sexually active as early as 9 years old; by the time they reach 14 years like Betty (pseudonym), they feel that they are grownups... My daughter Faith (pseudonym) tells me that some of her former primary school classmates now have three children yet she is still the way she was; I mean she has never been pregnant. She still wants to further her education and so she tells me that she cannot give birth at the moment until she achieves her education goals* – Nyasembo, Female Caregiver, Intervention

In sharp contrast, several adolescents from food insecure households in the control group were not able to stay in school

*… At times I’m sent away from school and I end up spending two weeks at home thus missing what the teachers taught in my absence; it then becomes difficult to catch up with my classmates and this greatly affects my performance … I spend time doing casual work instead of reading; I usually come back feeling very tired and so I end up sleeping without reading by books … I also get worried about my parents’ poverty status and this prevents me from focusing on my education because I really feel pained about our situation at home*. – Apondi, Adolescent, Control, 17 years

4. **Increased caregiver confidence discussing SRH with adolescents.**

*Shamba Maisha* emboldened the caregivers to speak about SRH and deter risky sexual behavior among their adolescents. Caregivers spoke with pride about having confidence to provide for and discuss sexuality with their adolescents.

*I warn them about taking money from men with confidence because I know that I can provide for their needs and that is why I know they have no reason to look for financial support from men. Before the intervention, I would still caution them, but I was not confident that they would listen to me because they knew that I could not afford to provide for all their needs. Therefore, they would be tempted to accept financial offers from men to meet their needs. –* Nyalengo, Female Caregiver, Intervention

In turn, because adolescents could see that their caregivers were more confident and able to provide, made the conscious choice to be more responsive to their caregivers and to avoid deviant behavior.

*I was rude before; she would speak to me then as I just stare at her like she was nobody. I also was lazy; I would be walking around when there were dirty clothes that needed to be washed. I just didn’t care … Before when my mother was talking to me, I felt that she didn’t deserve speaking to me; I felt that she had nothing or rather knew nothing to talk about. Later when she joined Shamba Maisha and started improving the family’s status through the work she was doing in her farm, I decided to change and give her time and also listen to the advice she was giving me because she now had something to offer. –* Adhiambo, Adolescent, Intervention, 17 years

However, this adolescent girl from the intervention group still maintained her relationship with her partner because she felt embarrassed to ask her mother to provide for things like sanitary towels.

*The desire was still there but not as much because our family finances improved after my mother joined Shamba Maisha. However, I still wanted to date a man who would give me money to buy personal items like sanitary towels so that I didn’t have to go back to my mother and ask for sanitary towels when I needed them. It is somewhat embarrassing to ask my mother for sanitary towels* - Awiti, Adolescent, Intervention, 19 years

## Discussion

Our data highlighted key mechanisms for how the *Shamba Maisha* agricultural and finance intervention improved adolescent sexual health including improved confidence in discussing SRH topics among caregivers, improved sexual relationship power equity and school attendance among adolescents in the intervention arm. In contrast, driven primarily by the need for food and basic needs such as sanitary towels, girls in the control arms described engaging in transactional sex, having multiple sexual partners, being unable to focus in school, and getting pregnant or acquiring HIV. The findings among girls in the control arm corroborate many qualitative and quantitative studies in Africa that have found that food insecurity is associated with low sexual relationship power and high sexual risk taking among women [4, 5].

Among adolescent girls, sexual agency encompasses the longing for sexual and economic freedom and the striving for sexual rights (including sexual relationship power equity), coupled with developing of strategic negotiating skills required to navigate the social, moral and narrative contexts they live in. By focusing on critical social determinants of adolescent SRH, the *Shamba Maisha* intervention developed and promoted household contexts that enabled girls to challenge the nuanced and complex power dynamics within their surrounding communities. Participants described ways in which the intervention may have improved their self efficacy, self agency, self confidence in decision making and action regarding partner selection, negotiation skills, communication, risky sex and even desiring to stay in school longer [42]. Our data extend existing research by (1) corroborating the mechanisms of food insecurity driving risky sexual behavior among adolescent girls in HIV-affected households, and (2) demonstrating the paths in which addressing both food and financial insecurity can facilitate the rejection of social norms around reciprocity, gender and complacent femininities.

Food secure households used the newly acquired income to pay for school fees and keep girls in school. Being in school was highlighted as one the main reasons adolescents were motivated to delay having sex. Several studies in Africa have shown that higher levels of education are associated with lower HIV risk and higher HIV testing acceptance in AGYW [43]. In addition, when adolescents have access to assets via an income generating intervention such as *Shamba Maisha*, their behavior, attitudes, hopes for the future, desire to stay in school, and overall health are influenced in a positive manner [13, 15, 44] *Shamba Maisha* also emboldened the caregivers to speak about SRH and encourage their adolescents to avoid risky sexual behavior. Caregivers spoke with pride about having newly found confidence to discuss adolescent sexuality, and adolescents in turn were more responsive to their caregivers.

Structural interventions addressing poverty and health have historically been highly compartmentalized and neglected aspects of food security in AGYW. Data from our study adds value to the existing literature on the design of structural interventions by providing evidence on the potential benefit of situating AGYW structural interventions within social/familial structures. Economic empowerment interventions with a classic microfinance model may fail with most vulnerable girls because some of their most pressing needs may not relate to physical health and safety. In this study, we noted that some adolescents and their families in the intervention group were not shielded from anxiety regarding future food access even though at present they had enough to eat. This finding is important in that often the psychological and social aspects of food security such as uncertainty and anxiety about food are often overlooked when designing food security interventions. It is therefore important that future structural interventions are intentional about integrating household level interventions with interventions that deliberately target engendered psychosocial pathways that may diminish the long-term effectiveness of structural interventions.

Our study has several limitations. As a result of recall bias, intervention participants may have looked back upon the intervention more favourably upon recollection. In addition, social desirability bias may have influenced the narratives of the participants who wanted to represent experiences with the intervention as favourably as possible to interviewers. To minimize this bias, we used experienced young local female researchers who were not involved in intervention delivery, and provided training on open-ended unbiased interview techniques. Lastly, the study was conducted predominantly among the Dholuo in southwestern Kenya, therefore cultural variations beyond this region may not be represented.

## Conclusions

Despite the growing interest in adolescent SRH by the global community, the intersecting problems of food insecurity, poverty, HIV acquisition risk and poor SRH outcomes for adolescents in sub Saharan Africa have not been adequately addressed. This study explored the perceived impact of and the pathways by which a household level income generating agricultural intervention may impact adolescents’ vulnerability to sexual risk taking. Our findings suggest that deploying the intervention at the household level may be an effective strategy to reduce sexual risk taking among adolescents. Secondly, this work also provides an innovative approach to address the intersections between adolescent sexuality, agricultural and socio-economic problems. Collaborations across these different sectors can contribute toward sustainable public health solutions to reduce HIV acquisition risk and poor SRH outcomes for adolescents in SSA. Our findings suggest that future household level interventions targeting HIV-affected adolescent girls should consider addressing food insecurity as part of an integrated package.

## Supplementary Information

**Additional file 1.** Adolescent Shamba Maisha interview guide- Intervention adolescents.

**Additional file 2.** Adolescent Shamba Maisha interview guide- Control adolescents.

**Additional file 3.** Adolescent Shamba Maisha interview guide- Caregivers.

## Data Availability

Data cannot be shared publicly because this study was conducted with approval from the Kenya Medical Research Institute (KEMRI) Scientific and Ethics Review Unit (SERU), which requires that we release data from Kenyan studies (including de-identified data) only after they have provided their written approval for additional analyses. As such, data for this study will be available upon request, with written approval for the proposed analysis from the KEMRI SERU. Their application forms and guidelines can be accessed at https://www.kemri.org/seru To request these data, please contact either the authors or the KEMRI SERU at seru@kemri.org

## Abbreviations

AGYW: Adolescent girls and young women
AIDS: Acquired immune deficiency syndrome
HIV: Human immunodeficiency virus
RCT: Randomized controlled trial
SSA: Sub Saharan Africa
STI: sexually transmitted infections
STD: Sexually transmitted diseases
SRH: Sexual reproductive health
UCSF: University of California San Francisco
