# Supplementary material for: The role of food security in increasing adolescent girls’ agency towards sexual risk taking: qualitative findings from an income generating agricultural intervention in southwestern Kenya
Source: BMC Public Health. 2021 Nov 6;21:2028. doi: 10.1186/s12889-021-12051-6 (PMC8572417; doi:10.1186/s12889-021-12051-6)
Supplement: Supplementary file 2 — Additional file 2. Adolescent Shamba Maisha interview guide- Control adolescents. [file 12889_2021_12051_MOESM2_ESM.doc]

# Qualitative Study Guide: Understanding the potential impact of a household level income generating agricultural intervention on adolescent sexual behavior and psychological well- being and the use of sexual and reproductive health and HIV services

# RESEARCHER NAME: ID:

**DATE:**

**Month Date Year**

**DISTRICT NUMBER: INTERVIEW NUMBER:**

**INTRODUCTION [AFTER GETTING CONSENT FROM PARTICIPANT]**

Thank you for meeting with me. We are talking to adolescent girls who have been living in a household that has been a part of *Shamba Maisha* program. We will begin by talking in general about adolescent girls and sexuality. We will then go deeper and talk about food security, wealth, health, mental health, parental communication and your household functioning. Lastly, we will ask about where you receive or can receive sexual reproductive health services.

It is okay if you do not know some details about the program- I am more interested in your overall experiences. There are no right or wrong answers to these questions and I am most interested in your detailed explanations and stories. In fact, I want you to speak most of the time and me to speak very little. This is your chance to tell me how you feel. Also, you are free to decline to answer questions that you do not feel comfortable answering. Please remember that anything you tell us is confidential and will not affect your involvement in receiving health care or at any health facility with the program in any way.

We would really like to protect your identity so kindly do not mention your name or any other person’s name when responding to the questions.

Do you have any questions about this project or the consent form?

[If not], OK…let’s go ahead and get started. Please stop me if you want to take a break, or if would like any part of interview to be unrecorded.

**Section A: ICEBREAKERS**

# [Note to interviewer: the purpose of this section is to begin engaging the participant in conversation and gain rapport.]

I would like to begin by asking several questions about you:

1. How are you doing today?
2. How old are you?
3. Have you ever attended school?
4. What is the highest standard/form in school that you have achieved?
5. Who in your household is in the Shamba maisha project?

**SECTION B: FOOD SECURITY**

# [Note to interviewer: the purpose of this section is to explore perceived changes in food adequacy, dietary diversity and frequency of meals.]

1. Over the past two years, can you talk about changes, if any, regarding your food situation? [PROBE FOR NARRATIVE STORIES]
   1. Probe on reasons for change in food situation if there was a change

**SECTION C: HOUSEHOLD WEALTH:**

# [Note to interviewer: This section seeks to explore adolescent’s perceptions on access to socially perceived household necessities e.g. toiletries, sanitary towels, clothes, and shoes, as well as school uniforms, stationery, and fees.]

1. Now I would like to hear about household items in your house such as toiletries, sanitary towels, clothes, shoes, uniforms, school stationery, and school fees. Tell me more about the availability of these necessities prior to joining Shamba Maisha?
   1. Probe on challenges with access to toiletries including sanitary towels, clothes, and shoes.
   2. [IF IN SCHOOL:] Probe on challenges in purchasing school uniforms, school supplies, and fees
   3. Probe on relying on charity/help from others to pay fees, staying in school, purchase school uniforms and stationery
2. Has your access to any of these items changed over the past 2 years?
   1. Probe on changes in need for her to work to supplement household income over the past two years
3. Over the past 2 years*,* have you noticed any changes in your general life at home or with friends? If so, please explain
   1. Probe on changes in family involvement (likelihood to spend more time in the household vs. visiting others, more social, spoken
   2. Probe on changes in increasing social networks e.g. had more friends, more visitors, more outgoing
   3. If there have been changes, probe on reasons for the changes

**(SKIP QUESTION IF PARTICIPANT HAS NOT BEEN IN SCHOOL IN THE LAST 2 YEARS)**

1. Over the past two years, have you noticed any changes in your schooling and education? Tell me about that
   1. Probe on changes in going, staying or skipping school (due to availability of fees or having not to work)
   2. Probe on changes in educational performance

If there have been changes in education, probe on reason for change.

1. In the last 2 years, can you speak of any changes you have noticed in your mental or physical health?
   1. Probe on changes in physical health
   2. Probe on changes in mood (happiness, symptoms of depression)
   3. If there have been changes, probe on reasons for change (ex: less stress, improved income, improved diet quality, improved family relationships etc…)
2. [ASK IF IN SCHOOL, OTHERWISE SKIP TO SECTION D] After you complete your secondary education, would you want to further your education? How else can we ensure that your education beyond secondary school is guaranteed? (Avoid answers like school fees payment)

**SECTION D: ENABLING CAREGIVING ENVIRONMENT**

# [Note to interviewer: This section seeks to explore concepts around involvement (i.e., spending time with and showing interest in the adolescent), quality of communication (i.e., parent empathy and conversation across situations). We are also interested in changes in parenting monitoring: 1) positive parenting, 2) consistent discipline, and 3) good supervision]

1. What are typical evenings/weekends like for your family?
   1. Probe on leisure activities that they involve the adolescent girl.
   2. Probe on activities that the adolescent girl is happy/unhappy/ interested/disinterested in.
2. When you are having issues with school or are having a bad day is there any family member who is primarily concerned? Tell me more about this.
3. How do you have discussions with your caregiver when you have any psychosocial issues like feeling low or afraid?
4. How does your caregiver or parent discipline/supervise you?
   1. Probe on how the caregiver checks on the way the adolescent girl(s) behaves or feels
   2. Probe on the caregiver’s style of discipline/supervision (how they reward good behavior and manage bad behavior, caution on any risky behavior)
   3. Probe on how the adolescent girl feels about the way they discipline?
   4. Probe on how the adolescent feels their caregiver’s style works, is there anything they would change?
5. Over the past 2 years, could you talk about changes, if any, you have experienced in how you relate with your caregiver/parent?
   1. Probe on changes in the way the caregiver monitors/supervises the adolescent
   2. Probe on changes in the way the caregiver disciplines the adolescent (e.g. stricter vs accommodating, physical vs verbal, withdrawing or giving affection or gifts.)
   3. Probe on changes in how the caregiver rewards good behavior and manages bad behavior, cautions on any risky behavior)
   4. Probe on changes in the communication between them and caregiver? (include quality and frequency, more or less involvement of third parties e.g. aunts or older sisters)
6. How would you like the environment at your home improved and made peaceful for you as an adolescent?

**SECTION E: SEXUAL HISTORY**

# [Icebreakers about community norms:]

1. Around when do girls start becoming sexually active in your community? By sexually active I mean engaging in any type of sexual activity (kissing, touching, or more) with a man.
2. How do girls usually meet these men? Where do they continue to meet while they are sexually involved?

**Thank you: I want us now to talk about *your* sexual experiences over the past two years.**

1. Have you had at least one person with whom you were sexually involved? [**IF SEXUALLY INEXPERIENCED, I.E. ANSWERED NO TO QUESTION 3, SKIP TO QUESTION 8**
   1. Probe: Have you ever been sexually involved with a man?
   2. Probe: What influences your partner choice/selection? (able to provide food (snacks), money, airtime or just access –availability- men are available during holidays)
2. Has there ever been a time where you received gifts for sexual activities or sex? What about a time where you were paid for sex or sexual activities?
3. Has there been a time when you had multiple partners? Or a time when you were seeing more than one person?

# [WHEN COMPLETE, SKIP TO QUESTION 5]

**QUESTIONS FOR SEXUALLY INEXPERIENCED GIRLS**

1. When do you think it is ok for females to start having sex?
   1. Probe: When is it too early to start having sex?
   2. Do you think girls in your community are pressured to have sex before they are ready? How or how not?
   3. Why do you think you haven't had sex yet?
   4. Do you feel ready? Why? Why not?
   5. Do you feel under pressure to have sex? From whom? How does this make you feel?
   6. Do you feel pressure not to have sex? From whom? How does this make you feel?
      - How have you resisted the pressure(s) to have sex?
      - Probe for the role of the families wealth status/security in enabling her avoid/delay sex
      - Probe for the role of the accessibility to food in enabling her avoid/delay sex
      - Probe for the role of parental monitoring and supervision in enabling her avoid/delay sex

# SEXUAL RISK TAKING [ASK FOR THOSE WHO ARE SEXUALLY ACTIVE—IF NOT SKIP TO SUB- SECTION E3: Risk perception; QUESTION 10]

**[Sub-section E1: Contraception]**

**Thinking now about the most recent sexual encounter over the past two years:**

1. Can you share with me what you know about contraception among young people?
2. Have you used contraceptives to prevent pregnancy? If so, which one(s)?
   1. Probe: Where were the methods of preventing pregnancy obtained from?
   2. Who provided them? How often did you use them?
   3. Which method did you use most commonly? Why did you use the method you chose?
   4. Was your partner involved in the decision making process? If so, how?
3. Did either of you have any problems / barriers to obtaining protection/contraception?
   1. Probe: What were they? Were they overcome? How? Why not?

# [Sub-section E2: Condoms][IF EVER SEXUALLY ACTIVE; OTHERWISE SKIP TO QUESTION 19:

# Did you use condoms with your last partner? Why did/did you not use condoms? In what instances did you not use condoms? How did you decide when and when not to use condoms? Who decided? Did you or partner influence when the condoms were not to be used?

1. Have your opinions of about use of condoms changed at all in the last two years? If so, how? What do you attribute the change in use of condoms to? Probe on whether family participation in SM had any impact.

# [Subsection E3: Risk perception

1. In general, do you consider yourself to be at risk of HIV, STI or pregnancy?
   1. Probe: why or why not?
   2. Probe: Have you ever been pregnant? What happened with the pregnancy? How did you feel? How did other people react? Did it change your behaviour?
   3. Probe: How would you feel if you found out you were pregnant? What would you do? Why?
2. Have you had a STI or symptom(s) of an STI? Have any of your partners had a STI or symptom that you know of? What happened? What did you do? How did you feel? How did this change your behavior, if at all?
3. Do your friends and other girls of your age commonly get infected with STIs in this community?
   1. Probe: Why do you think so?
4. What influences sexual behavior among girls of your age in this community?
   1. Why do you think these factors influence sexual behavior? [PROBE FOR EVERY FACTOR GIVEN]
5. Have you ever had an HIV test? Have any of your partners had an HIV test? Why? Why not?

**SECTION F: SEXUAL REPRODUCTIVE HEALTH CARE SEEKING**

# [Interview note: The purpose of this section is to learn more about where adolescent girls access HIV, STI and pregnancy testing, condoms or family planning methods.]

**Ok, now I’d like to ask about one more topic area…**

1. Can you list for me all the places young women are able to visit and people they can talk to, to find out about sex, contraception, PrEP, and/or STIs?
2. Have you ever gotten information from one of these places?
3. What do you think are the most important features of a sexual health service for young people?
   1. Probe: What do you think are the essential elements of a service?
   2. Probe: What will make young people go?
   3. Probe: Are there differences in the needs of young men and women? How can they both be provided for?
   4. Probe: What barriers are there for accessing these services?
4. Where do you think adolescent and young women’s sexual health services should be held (location)? Why? Who should provide the information and advice?
5. How do you think the services in your locality could be improved upon? What do adolescent and young women in your area need?

**SECTION G: GROUP DYNAMICS**

1. Having seen how your guardian/parent was involved in the intervention and how that impacted his/her life and that of your household in general; how do you think the intervention could made more beneficial for you and any other adolescent girls?
   1. Probe for possible youth group meetings? How can this be achieved? Possible venues for the meetings?
   2. Probe for possible topics to be discussed?
   3. Probe for the social networks they would like to build from the group meetings and their importance?
   4. Probe for other activities they would want to be included in the group meetings?

**SECTION G: CONCLUSION**

1. Are there any other topics that you would like to discuss?
2. Is there something you started to say about any topic that you did not get to elaborate on?
3. May I read through this interview guide quickly and make sure we have spoken about everything?

Thank you very much for your time. Do you have any questions about this interview before we end today?

# INTERVIEWER’S OBSERVATIONAL NOTES (Please write here):
