# Supplementary material for: The role of food security in increasing adolescent girls’ agency towards sexual risk taking: qualitative findings from an income generating agricultural intervention in southwestern Kenya
Source: BMC Public Health. 2021 Nov 6;21:2028. doi: 10.1186/s12889-021-12051-6 (PMC8572417; doi:10.1186/s12889-021-12051-6)
Supplement: Supplementary file 3 — Additional file 3. Adolescent Shamba Maisha interview guide- Caregivers. [file 12889_2021_12051_MOESM3_ESM.doc]

**Qualitative Study Guide: Understanding the potential impact of a household level income generating agricultural intervention on adolescent sexual behavior and psychological well-being and the use of sexual and reproductive health and HIV services.**

**Please collect PAGE 1 for each interviewee**

| **RESEARCHER NAME:__________________ ID:**  **DATE:**  **Month Date Year**  **DISTRICT NUMBER: INTERVIEW NUMBER:** | . |
| --- | --- |

**INTRODUCTION**

Thank you for meeting with me. We are talking to women and men who have been a part of Shamba Maisha and have adolescent girls living in their households. Today I would like to hear more about the potential impact of a household level income generating agricultural intervention on adolescent behavior, psychological well-being and their uptake of sexual and reproductive health and HIV care services. We will begin by talking in general about adolescent girls and sexuality. We will then go deeper into your experience with the *Shamba Maisha* project as a study participant and talk about food security, wealth, health, parental communication and how your household functions. Lastly, we will ask about where the adolescent girl in your household receives or can receive sexual reproductive health services. I am also interested in any recommendations/suggestions you have for on how we can improve the *Shamba Maisha* project so that it benefits adolescent girls like the one in your household.

Don’t worry if you can’t remember some details of the program - I am more interested in your overall experiences. There are no right or wrong answers to these questions and I am most interested in your detailed explanations and stories. In fact, I want you to speak most of the time and me to speak very little. This is your chance to tell me how you feel. Also, you are free to decline to answer these questions. Please remember that anything you tell us is confidential and will not affect your involvement in receiving health care care or with the program in any way.

We will collect the information, keeping participants anonymous. When responding to questions, please do not identify yourself or anyone else by name.

Do you have any other questions about this project or the consent?

[If not], OK…let’s go ahead and get started. Please stop me if you want to take a break, or if would like any part of interview to be unrecorded.

**Section A:**  **ICEBREAKERS:**

**[Note to interviewer: the purpose of this section is to begin engaging the participant in conversation and gain rapport. ]**

I’d like to begin by asking several questions about you:

1. How are you doing today?
2. How many children do you have?
3. Do your children attend school?

**SECTION B: FOOD SECURITY**

**[Note to interviewer: the purpose of this section is to explore perceived changes in food adequacy, dietary diversity and frequency of meals.]**

1. To get started, I would like to hear first about your family’s food situation. Tell me more about your food situation prior to joining *Shamba Maisha*? **[FOR CONTROL PARTIPANTS, ASK THIS QUESTION ABOUT CURRENT FOOD SITUATION]**
   1. Probe on challenges with access to food, and food shortages
   2. Probe on challenges with diet quality and diversity of available foods
   3. Probe on relying on charity or others for food
2. Since your household joined *Shamba Maisha*, could you talk about changes, if any, you have experienced regarding your own food situation? [PROBE FOR NARRATIVE STORIES]
   1. Probe on changes in number of meals eaten in a day
   2. Probe on changes in frequency of food shortages, particularly during dry season
   3. Probe on changes in diet/variety of foods served and if changes limited to dry season
   4. Probe on changes in distribution of food among household members (especially to the adolescents in the house)?
   5. Probe on reasons for change in food situation
   6. Probe on other continued food-related challenges despite being in the program

**FOR INTERVENTION PARTICIPANTS ONLY [CONTROLS SKIP TO SECTION C]:**

1. How do you think the Shamba Maisha intervention affected availability of food for your adolescent girl(s)?
   1. Probe on changes in distribution of food based on gender (male and female adolescents)
2. How do you think the Shamba Maisha intervention could be improved to further improve access to food for adolescent girls such as the one who is living in your household?

**SECTION C: HOUSEHOLD WEALTH:**

**[Note to interviewer: This section seeks to explore caregiver’s perceptions on access to socially perceived household necessities e.g. toiletries, sanitary towels, clothes, and shoes, as well as school uniforms, stationery, and fees.]**

1. Now I would like to hear about household items in your house other than food such as toiletries, sanitary towels for girls, clothes, shoes, uniforms, school stationery and school. Please tell me more about availability of these household items prior to joining *Shamba Maisha*?
   1. Probe on challenges with access to toiletries including sanitary towels, clothes, and shoes
   2. [IF GIRL IS IN SCHOOL] Probe on challenges in purchasing school uniforms, school supplies, and fees, including relying on charity
   3. Probe on challenges with quality and variety of available toiletries including sanitary towels, clothes, and shoes, as well as school uniforms, stationery, and fees
   4. Probe on relying on charity or others for purchasing toiletries including sanitary towels, clothes, and shoes for household in general
2. Since your household joined *Shamba Maisha*, could you talk about any changes you have experienced in terms of access and purchase of toiletries, sanitary towels for girls, clothes, shoes, uniforms, school stationery, school fees? [PROBE FOR NARRATIVE STORIES]
   1. Probe on changes in income to ensure basic needs for adolescent girls
   2. Probe on changes in income to ensure adolescent girls were kept in school and not missing school due to lack of fees, uniform, stationery, sanitary towels
   3. Probe on changes in proportion of income within the family allocated to adolescent girls
   4. Probe on changes in having to give up basic needs due to a tight financial situation
   5. Probe on changes in relying on charity/help from others compared to before intervention
   6. Probe on other possible explanations for the changes
3. Since your household joined *Shamba Maisha,* could you talk about any changes you have noticed in you the life your adolescent girl. I am talking about life in general including her friends, her attitudes, behaviour or how she interacts with people including you?
   1. Probe on changes in distribution of assets within the family (younger children vs adolescents vs adults)
   2. Probe on changes in need for the adolescent to work to supplement household income before joining the program
   3. Probe on changes in family involvement (likelihood for her to spend more time in the household vs. visiting others, or becoming more social and outspoken)
   4. Probe on changes in social networks (e.g. had more or less friends, more or fewer visitors, more or less outgoing)
4. [IF HAS ADOLESCENT GIRL IN SCHOOL] Since your household joined Shamba Maisha, can you speak about any changes you have noticed in your adolescents involvement in schooling?
   1. Probe on changes in attendance (due to availability of fees or having not to work)
   2. Probe on changes in educational performance

**[FOR INTERVENTION PARTICIPANTS ONLY; CONTROL PARTICIPANTS SKIP TO SECTION D]**

- 1. Did *Shamba Maisha* have an effect on your adolescent girls’ education in any way? If so, please describe how.
  2. Probe on mechanisms for change (e.g. more income for school fees, better concentration due to less stress, less food insecurity, or reduced need to work to stay in school; improved family relationships)

1. Since you joined *Shamba Maisha,* can you speak of changes, if any, you have noticed in the behavior and health of your adolescent girl(s)?
   1. Probe on changes in mood (happiness e.g. she laughs more, less moody)
   2. Probe on changes in physical health
   3. Probe on mechanisms for change (e.g. less stress, improved income, improved diet quality, improved family relationships, etc)
2. Based on what you just said about the impact of *Shamba Maisha* on your adolescent girls’ life, how do you think the *Shamba Maisha* intervention could be further improved to help your adolescent’s belief in her own ability to succeed in school and in life in future?
   1. Probe on ways SM can further improve schooling
   2. Probe on ways that SM can further improve the adolescent girl’s physical health
   3. Probe on ways that SM can further improve the mood and mental health of the adolescent girl

**SECTION D: ENABLING CAREGIVING ENVIRONMENT**

**[Note to interviewer: This section seeks to explore concepts around involvement (i.e., spending time with and showing interest in the adolescent), quality of communication (i.e., parent empathy and conversation across situations). We are also interested in changes in parenting monitoring: 1) positive parenting, 2) consistent discipline, and 3) good supervision]**

1. What are typical evenings/weekends like for your family?
   1. Probe on leisure activities that involve the adolescent girl(s)?
   2. Probe on activities that the adolescent girl(s) are happy/unhappy/ interested/ disinterested with?
2. When your adolescent girl(s) is having issues with school or are having a bad day is there any family member who is primarily concerned? Tell me more about this.
3. Can you describe to me how your adolescent girl lets you know when she is feeling low or afraid? Can you describe to me to a time when she came to you with these feelings?
4. As a caregiver, how do you discipline or supervise your adolescent girl(s)
   1. Probe on how they check on the way their adolescent girl(s) behave or feel
   2. Probe on the style of discipline/supervision (how they reward good behavior and manage bad behavior, caution on any risky behavior)
   3. Probe on whether they think it is effective
   4. Probe on how they think the adolescent girl(s) feels about the way they discipline?
   5. Probe on how confident they feel their style works, is there anything they would change
5. Since you joined *Shamba Maisha*, could you talk about any changes you have experienced in how you relate with your adolescent girl(s)
   1. Probe on changes in the way they monitor the adolescent girl
   2. Probe on changes in the way they discipline the adolescent girl (e.g. stricter vs. accommodating, physical vs. verbal, withdrawing or giving affection or gifts.)
   3. Probe on changes in how they reward good behavior and manage bad behavior, caution on any risky behavior)
   4. Probe on changes in the communication between them and adolescent girl(s)? (e.g. quality and frequency, more or less involvement of third parties e.g. aunts or older sisters
6. **INTERVENTION PARTICIPANTS ONLY [FOR CONTROL PARTICIPANTS, SKIP TO SECTION 5]:** How, if at all, do you think the *Shamba Maisha* intervention has affected your relationship with your adolescent girl?
   1. Probe on mechanisms for change (ex: reduced stress from less food insecurity, more household wealth and assets, increased time spent together e.g. working on the farm together or parents doing less side-hustles)
7. What other kind of intervention would you recommend to be paired with Shamba Maisha that would be improve your relationship with your adolescent girls?
   1. Probe – direct counseling of parenting and adolescents at home, parenting classes on how to handle adolescents, communication classes, community or school based programs, other income generating activities, etc.

**SECTION E: COMMUNICATION ABOUT SEX**

1. Since your household joined *Shamba Maisha,* have you had conversations with your adolescent girls about sexual reproductive health issues? Why or why not?
   1. Probe: was this happening before *Shamba*?
   2. [**For those who discuss** sexual and reproductive health **issues with their girls:**] Can you talk to me a little more about the types topics you discussed with her?
      1. Probe: did you discuss menstruation and bodily changes?
      2. Probe: did you discuss appropriate sexual behavior?
      3. Probe: did you discuss pregnancy and childbirth or how girls and women become pregnant?
      4. Probe: did you discuss contraception and condom use?
   3. Can you talk to me about how easy/hard it is to discuss these topics?
   4. Which topics did you consider a priority in discussing? Why?
   5. Probe specific messages delivered on this topic.
2. Could you talk about your own comfort level in talking about any of these topics with your adolescent girl?
   1. Probe on whether and how this (comfort level) has changed since the start of the study.
   2. Probe on whether comfort level changes as topics change (i.e more comfort talking about one over other, and why)
   3. Probe on frequency of conversations and variety of topics.
3. Where do adolescent girls learn about contraception, condom use, and PrEP? Probe, if needed:
   1. Extended family members/adults (who specifically?)
   2. Initiation counselors at school
   3. Friends/same-age peers
   4. Health workers (what type specifically?)
   5. Media (e.g., radio, magazines, newspapers, TV, advertising, billboards, internet, etc.)
   6. Probe: are the sources they list good sources of information? Why or why not?
4. Is childbearing among girls under 18 common in your community? Do you think this influences your own girl’s likelihood of getting pregnant when under 18?

FOR INTERVENTION PARTICIPANTS:

1. How, if at all, do you think the *Shamba Maisha* intervention has affected your communication with your adolescent girl?
2. Probe about changes in communicating about bodily changes, sexual health, sexual behavior including avoiding pregnancy and having safer sex or sexual relationships with your adolescent girl?
3. Probe on mechanisms such as reduced stress from less food insecurity, more household wealth and assets, increased time spent together e.g. working on the farm together or parents doing less side-hustles)
4. What other kind of intervention would you recommend to be paired with Shamba Maisha that would be improve your communication with your adolescent girls about sexual and reproductive health issues?
   1. If needed, give examples, such as direct counseling of parenting and adolescents at home, parenting classes on how to handle adolescents, communication classes, community or school based programs, community health workers, etc.

**SECTION F: SEXUAL HISTORY AND SEXUAL REPRODUCTIVE HEALTH SERVICES**

**[Icebreakers about community norms:]**

1. Around when do girls start becoming sexually active your community? By sexually active I mean engaging in any type of sexual activity (kissing, touching, or more) with a man.
2. How do girls usually meet these boys or men? Where do they meet while they are sexually involved?
3. Based on your own knowledge or perception of your adolescent girl, is your adolescent girl currently in a romantic relationship with a boy or man?
   1. Probe: If yes, please tell me what you know about the relationship of your adolescent girl with this person. When did it begin? How do you feel about her being in a relationship? What do they do together?
4. Were there any sexual practices that your adolescent was engaging in before *Shamba Maisha* that you did not feel good about and they were able to stop? Tell me about that.
   1. Probe on mechanisms such as reduced stress from less food insecurity, more household wealth and assets, increased time spent together e.g. working on the farm together or better household communication and environment)

**As I we finish, lets talk about sexual reproductive health services**

1. What other kind of interventions would you recommend to be paired with Shamba Maisha that would increase adolescent girls' utilization of needed sexual and reproductive health services?

**SECTION G: CONCLUSION**

1. Are there any other topics that you would like to discuss?
2. Is there something you started to say about any topic that you did not get to elaborate on?
3. May I read through this interview guide quickly and make sure we have spoken about everything?

Thank you very much for your time. Do you have any questions about this interview before we end today?

**INTERVIEWER’S OBSERVATIONAL NOTES (Please write here):**
